# Prevalence and influencing factors with knowledge, attitude, and practice toward anemia among school-going adolescent girls in rural Bangladesh

**DOI:** 10.1371/journal.pone.0313071

**Published:** 2024-11-18

**Authors:** Md Jiaur Rahman, Md Moshiur Rahman, Mohammad Habibur Rahman Sarker, Masayuki Kakehashi, Miwako Tsunematsu, Mohammad Ali, Ashir Ahmed, Mohammad Delwer Hossain Hawlader, Yoko Shimpuku

**Affiliations:** 1 Department of Health Science, Graduate School of Biomedical and Health Sciences, Hiroshima University, Hiroshima, Japan; 2 Nutrition and Clinical Services Division, International Centre for Diarrhoeal Disease Research, Bangladesh, Dhaka, Bangladesh; 3 Department of Medicine, Comilla Medical College, Comilla, Bangladesh; 4 Department of Advanced Information Technology, Graduate School of Information Science and Electrical Engineering, Kyushu University, Fukuoka, Japan; 5 Department of Public Health, School of Health and Life Sciences, North South University, Dhaka, Bangladesh; National Research Centre, EGYPT

## Abstract

**Background:**

Anemia is a major public health concern; however, research on anemia and its contributing variables is scarce. To address the gap, we investigated the prevalence and associated factors of anemia in school-aged adolescent girls in rural Bangladesh.

**Methods:**

We conducted a cross-sectional study in two secondary schools at Chandpur, Bangladesh, from January to April 2022. We randomly selected 422 adolescent girls based on prevalence rates from existing research to ensure reliable estimates and a robust study representation. We performed interviews with a semi-structured questionnaire after receiving ethical permission and written informed consent. In addition, physical examination and anthropometric measurements were done, followed by blood testing to identify anemia. Blood hemoglobin was measured using a spectrophotometric method, and quality control was ensured by validated results with control samples. Univariate with multiple logistic regression was employed for analysis to find the association between anemia and all other variables that were adjusted to control the potential effect of confounding variables.

**Results:**

The overall prevalence of anemia was 37.7% (mild- 33.8% and moderate- 3.9%). In the univariate analysis having a working mother (OR 3.84; 95% CI 1.31–11.26), undernutrition (OR 2.40; 95% CI 1.27–4.52), and irregular lunch consumption (OR 2.15; 95% CI 1.00–4.60) were associated with anemia. Conversely, being a user of a slab latrine (OR 0.61; 95% CI 0.40–0.93) and not weekly consumption of pure milk (OR 0.62; 95% CI 0.41–0.92) were negatively associated with anemia. In multivariate logistic regression analysis, associated factors for prevalent anemia were working mother [adjusted odds ratio (aOR) 7.65; 95% CI 1.97–29.50], slab latrine user (aOR 0.46; 95% CI 0.26–0.79), and irregular lunch consumption (aOR 2.92; 95% CI 1.21–7.03).

**Conclusion:**

The prevalence of anemia among adolescent girls in rural Bangladesh remains high with undernutrition. Anemia is associated with working mothers, slab latrine users, and irregular lunch consumers within the studied population.’ Thus, policymakers may promote school-based nutritional education and lunch programs with iron supplementation and improve sanitary facilities.

## Introduction

Anemia is a widespread nutritional deficiency disorder that poses a significant public health threat globally, particularly in low and middle-income countries (LMICs) [1,2]. Anemia affects an estimated 1.62 billion people worldwide; however, children, adolescents, reproductive-age women, and pregnant women are more susceptible to developing anemia [3]. Adolescence, defined as the period between the ages of 10 and 19 years, is a crucial period for maturation and reproductive changes, demands adequate iron consumption for optimal growth [4]. According to UNICEF, the global crisis of malnutrition and anemia affects vulnerable adolescent girls living in developing countries [5]. Approximately one-sixth of the world’s population is adolescents, with almost 90% of them residing in LMICs, where there are high disease burdens, inadequate nutrition, poor economics, and low education levels [6,7]. The prevalence of anemia among adolescents in LMICs ranges from 13.4% to 62.9% [4]. However, anemia, which is an alarming public health issue that can hinder national growth, is typically ignored in developing nations [8].

Adolescent girls are particularly vulnerable to anemia due to menstrual blood loss, high rates of infection and worm infestation, and inadequate iron intake [4]. The risk factors for developing anemia include a lack of nutritional knowledge, poor economic status, dietary habits, meal skipping, inadequate dietary & iron intake, poor personal hygiene, and infectious diseases [9]. Effectively handling and reducing the risks associated with anemia holds immense significance, as it has been documented to be essential for ensuring healthy growth for becoming a future healthy mother [10]. On the other hand, proper nutritional knowledge, attitudes, and practices (KAP) are more linked to improved health behaviors, which may contribute to prevention anemia and other health issues related to risk factors in adolescents [11].

Bangladesh is a developing country in South Asia with a population of 165.1 million, the vast majority of whom reside in rural areas [12]. However, most studies on anemia in adolescent girls have been conducted in urban areas, and the prevalence ranges from 23% to 51.6%. [3,13]. A systematic review on anemia in adolescent girls in Bangladesh identified seven studies conducted primarily in the capital city of Dhaka region based until July 2022. No peer-reviewed research on anemia in school-going adolescent girls in rural areas was found [14]. Two studies were identified on schoolgirls, but both were conducted two decades ago in the Dhaka district of Bangladesh’s major metropolis, lacking an accurate representation of rural areas [15,16]. Furthermore, nutritional KAP research lags among school-aged adolescents in rural Bangladesh. Therefore, we designed and implemented the school-based screening program to gather information on the prevalence and associated factors of anemia, as well as KAP towards anemia among adolescent girls in rural Bangladesh.

## Materials and methods

### Study design and settings

A school-based epidemiological cross-sectional study was conducted from January to April 2022 in Chandpur district, which is a part of the Chittagong division of Bangladesh. The Chandpur district is 106 km away from the capital city of Dhaka, and the total population is 2,635,748; among them literacy rate is 78.05% [17]. The study was conducted in two schools which are located in different *Unions* of the Sadar sub-district of Chandpur. (1) The Baburhat School & College school is situated in the Sadar *Pouroshova*, a combined school for boys and girls. The total number of students at this school was 1676, among them 62% were girls. (2) The Uttar Shahatali Zobaida Girls High School is in the Shah Mahmudpur *Union* and is exclusively for girls, with a total of 310 students.

### Study population

Adolescent girls of class/grades 6–10, aged 10 to 19 years old, from Baburhat High School & College and Uttar Shahatali Zobaida Girls High School, who completed a written informed consent with their legal guardians were eligible to participate in this study. However, participants who were physically and mentally ill at the time of enrollment were excluded from the study.

#### Sampling procedure

First, student information including name, identification number, and grade was obtained from the register books of each school. A stratified random sampling technique was performed based on student classes/grades into distinct strata for recruiting the participants. The study participants were stratified into five groups (class 6 to 10), and a separate stratum was considered for each class. After inputting student information into Microsoft Excel, a computer-generated random number sequence was used to select participants with probability proportional to the size of students in each class/grade of school. Through this sampling technique, we ensured a fair representation from each stratum because larger classes had a higher chance of enrolling more participants.

#### Sample size estimation

The existing literature review found that the prevalence study used an estimated prevalence rate for their study sample size calculation [2]. For our sample size calculation, an estimated anemia prevalence was carefully considered, which is an important parameter. Our literature review revealed that anemia prevalence exhibits variations according to area/region, but nationwide prevalence adequately represents all areas including rural settings. Due to this consideration, we chose the estimated anemia prevalence of 51.6% in adolescent girls in Bangladesh from a nationwide cross-sectional study [3]. To estimate the sample size, the following formula was used: n = (z)^2^ p(1-p)/d^2^. Here, n represents the sample size, z represents the level of confidence (which was set at 95%, resulting in a value of 1.96), p represents the estimated proportion of prevalence of anemia in adolescent girls (which was 0.516), and d represents the tolerated margin of error (which was set at 0.05). Based on this formula, the calculated sample size was 384. Considering the 10% drop-out rate due to unavailability, absence, or refusal to provide blood samples, the final sample size was 422 and 211 from each school.

#### Data collection of the study participant

Trained community health care workers (CHWs) conducted survey questionnaire interviews and collected anthropometric data after obtaining written consent from participants’ legal guardians. Age, student grade, menstrual history, hygiene management, sanitation facility, dietary habit, and lifestyle information were collected during the interviews employing a field-tested structure questionnaire. In addition to participants’ data, information on parents’ occupations, education, and monthly family income was acquired. Following the interview, CHWs assessed participants’ height (in cm), weight (in kg), waist circumference (in cm), hip circumference (in cm), and mid-upper arm circumference (MUAC) (in cm). In addition, the CHWs applied an additional field-tested questionnaire created by the "Food and Agriculture Organization of the United Nations (FAO)" to evaluate anemia related KAP [18]. The KAP questionnaire comprised 21 questions (knowledge—8, Attitude—6, and practice -7). The details of the KAP questionnaire are described in Table 2.

### Questionnaire validity and reliability

To conduct this study, the researcher developed a part questionnaire based on previous studies [2,3,4,11]. We carefully reviewed the exiting studies according to considering adolescent population group, school-going adolescent, rural context, cross-sectional study which align with our study objective. Also, the survey questions were selected based on reviewed studies relevance to our research aims and the specific requirements of our study participants. The KAP questionnaire was adopted from FAO, which is previously developed, and it was implemented in different regions over the world for the scientific research among the adolescent’s girl to access KAP. The knowledge and practice parts of the questionnaire were modified according to context of local culture, food habits and food availability. Then the translation to Bangla was performed according to forward-backward translation to ensure linguistic accuracy and cultural equivalence of the questionnaire. A physician and a university lecturer, both native speakers of Bangla and fluent in English, translated the questionnaire into Bangla first. The translated Bangla versions were compiled, and a single Bangla forward version was created. This forward version was then translated back into English by a professional translator with experience in medical translation and by one medical doctor who had not been involved in previous steps. The back-translated versions were then compiled and compared by the researcher, and all four versions were submitted to the public health professional expert committee that was formed for the validation study. The expert committee developed the questionnaire.

The questionnaire was initially in English but later translated into the local language Bangla. To enhance the face validity of the questionnaire, a pre-test survey was conducted among a randomly selected 5% proportion of study participants from the designated school. In the pretest survey, carefully examined the participant’s suggestions and comments, addressing refining local language, clarity of the questionnaire items, ambiguities, and articulating the local fruit names with food habits. The feedback from the participants was refined one by one to develop and finalize the questionnaire while ensuring the questions are clear and culturally appropriate for the study context. Afterward, to assess the reliability of the questionnaire, the test-retest method was utilized. The targeted participants were invited to a rest-retest survey with the same group of participants at two-week intervals. We performed Cohen’s Kappa coefficient analysis for the reliability of our questionnaire. However, participants who were involved in the validity and reliability process were excluded from the final survey.

### Sample collection of the study participant

The blood samples were collected following a standardized procedure guideline to ensure consistency and accuracy in the testing process. The samples were collected in accordance with the World Health Organization (WHO) guidelines [19] for sample preservation to ensure that the samples remained viable for testing purposes. Experienced medical technologists collected blood samples from the study participants in school settings. Due to the accessibility and minimal discomfort of the participant, the venipuncture site was selected median cubital vein and sterilized the area before blood was taken. To minimize potential contamination, use a one-time applicable sterilize pad, butterfly needle, gloves, and hand sanitizer during the blood collection. The blood samples (2 ml each individual)) were collected in the Ethylenediaminetetraacetic acid (EDTA) anticoagulant-containing vacuum tubes which were leveled with the unique barcode ID for each participant. An ice cooler box (2°C to 8°C temperature) was used to ensure stability of the blood samples during transportation which enhances the reliability and accuracy of hemoglobin test results. Within four hours samples were transferred to the laboratory for investigation. Furthermore, a register notebook was maintained to document the details of each sample, including its serial number, the time and date of when it was collected, and method of transportation.

#### Laboratory investigation

Lab Aid Diagnostic Center is a trusted and leading healthcare institution in Bangladesh, offering comprehensive diagnostic services through multiple branches across the country and delivering high-quality laboratory services. The laboratory investigations were performed at the “Lab Aid Diagnostic Center, Comilla” using Sysmex automated hematological analyzer (Model: XN-550). The spectrophotometric method was used to measure blood haemoglobin level from the whole blood sample. According to the manufacturer’s guidelines quality control ensures that running the control samples with known hemoglobin levels for confirming the analyzer gives precise and accurate results. The laboratory maintained international Standard Operating Procedures (SOPs) considering individual sample identification using unique barcodes to prevent mix-up, storage of the samples at 8 2°C to 8°C temperature, analyzer calibration, quality control ensures, and test results are prepared both hard and soft copies.

### Definition

#### Anemia

According to WHO, anemia was defined as having a haemoglobin level below a certain range. For adolescent girls aged 10–11 years, a haemoglobin level < 11.5 g/dl is considered anemic, with levels between 10–11.4 g/dl, 7.0–9.9 g/dl, and < 7.0 g/dl considered mild, moderate, and severe anemia, respectively. Non-pregnant adolescent girls aged 12–19 years with a haemoglobin level < 12 g/dl considered anemic, with levels between 10.0–11.9 g/dl, 7.0–9.9 g/dl, and < 7.0 g/dl considered mild, moderate, and severe anemia, respectively [20,21]. The literature review found that developing countries including Bangladesh used WHO cutoff values to define and categorize adolescent girls’ anemia. [2,3,11].

#### Undernutrition

Undernutrition of adolescent girls’ was defined as a MUAC of ≤ 18.5 cm and ≤ 22 cm for individuals aged 10–14 years and 15–19 years, respectively [22].

#### Obesity, overweight, and underweight

According to WHO, adolescents are underweight if their Body Mass Index (BMI) Z-score is < -2SD. Conversely, adolescents are considered overweight if their BMI Z-score is > +1SD, and they are categorized as obese if their BMI Z—score is > +2SD [23]. The existing literature revealed that over the world widely used WHO’s BMI Z–score for adolescent girls Obesity, overweight, and underweight determination [2,3].

#### Abdominal obesity

Abdominal obesity is defined as having a waist circumference < 71 cm [24].

#### Illiterate

Participants whose parents had not received any formal education were considered illiterate [25].

#### Irregular menstruation

Menstrual cycle is regular, with an average length of 28 days (the gap between periods starting), and irregular, with cycles lasting longer than 35 days or shorter than 21 days compared to the usual cycle [26].

#### Dependent variable

The dependent variable is the outcome measure variable, and anemia was the dependent variable in this study when the hemoglobin level is <12 g/dl for adolescents aged 10–11 years and <11.5 g/dl for adolescents aged 12–19 years considered anemia.

#### Independent variables

Independent variables are factors that affect the effect of the dependent variable. In this study, independent variables were age group, schools, student grade, family members, sibling, mother’s education, mother’s occupation, father’s education, father’s occupation, monthly family income, menstruation cycle, use of during the menstruation, use of toilets, weekly drink pure milk, weekly eat green leafy vegetables, weekly eat chicken, weekly eat red meat, eat breakfast, eat lunch, eat dinner, BMI—Z–score, MUAC, waist circumference, knowledge, attitude and practice.

### Statistical analysis

The IBM SPSS statistical software version 25.0 (IBM Co., Armonk, NY, USA) was used to analyze the data. Descriptive analysis presented frequencies and percentages for categorical variables, while mean and standard deviation (SD) were used to express continuous variables. The prevalence of anemia was considered the outcome variable, while age, student grades, parent’s education, monthly family income, dietary habits, knowledge score, attitude score, and practice score were considered exposure variables. For continuous variables, either a t-test or Mann-Whitney U test was conducted, depending on the data distribution. To investigate the associations between outcome and exposure variables of categorical data, the chi-square test was performed. Furthermore, multivariate logistic regression analysis was conducted, with the non-anemic considered as the reference group to determine the aORs and evaluate the strength of associations. To address confounding factors during multivariate logistic regression analysis, relevant covariates were carefully identified through literature review. The aim of inclusion was to mitigate potential confounding effects, ensuring a more accurate assessment of the relationship between the independent variables and the outcome. The statistical significance and impact of covariates were carefully considered, and the final analysis was adjusted for these factors. A statistical significance level of < 0.05 was considered.

#### Ethics statement

Ethical approval was obtained from the Institutional Review Board/Ethics Review Committee (IRD/ERC) of North South University Bangladesh (approval No: 2021/OR-NSU/IRB/1102) for this study, which is being conducted in accordance with the Declaration of Helsinki [27]. We submitted the detailed research protocol including the English and Bengali versions of the survey questionnaires and the informed consent form for their review and approval. The review committee assessed the comprehensibility and clarity of the protocol to meet ethical standards. According to the review committee’s feedback modification was made and the final protocol was approved before field implementation. In all phases of our research follow strictly adhered to ethical guidelines for the rights and well-being of the participants. We assured that there is no minimal risk of participants, and strictly maintained the privacy of personal information that was kept separate from research data, anonymized data present with specific researcher data handling. We also detail mentioned in the assent and consent form regarding the study aim, privacy, data handling, duration, voluntary participation and right to withdraw at any time. Also, after obtaining ethical approval, we shared a hard copy of the study procedure with the local Chandpur district education officer and school teachers. They reviewed the document and had a verbal discussion with the research team regarding the study procedure. After a clear understanding of the objectives of the study, they given permission to implement the research project in the schools (S1 File).

#### Informed consent

Written assents were obtained from participants, as well as written consents from the participants’ legal guardians, at the time of enrollment. Before this, the research team visited the schools and then described the study procedure, objectives, types of data with collection procedure, data privacy, voluntary participation and right to withdraw at any time, and duration of completing the process among each class of students with the help of teachers. The informed assent form was distributed to students who agreed to participate in the study by the research team. Following the completion of the assent form, the consent form was distributed. Participants took the form home to inform about the study with their guardians and obtained signatures from those who consented to allow their daughter to participate. A contact information with mobile number of the research team was given in the consent form, for the replying to the guardian’s quarries regarding the study. They were assured that the information obtained from them would not be disclosed. However, they were told about the use of data for assessment and the use of findings to improve patient care activities, as well as publication, without exposing their identity or identification (S1 File).

## Results

### Sociodemographic characteristics

**Fig 1:**
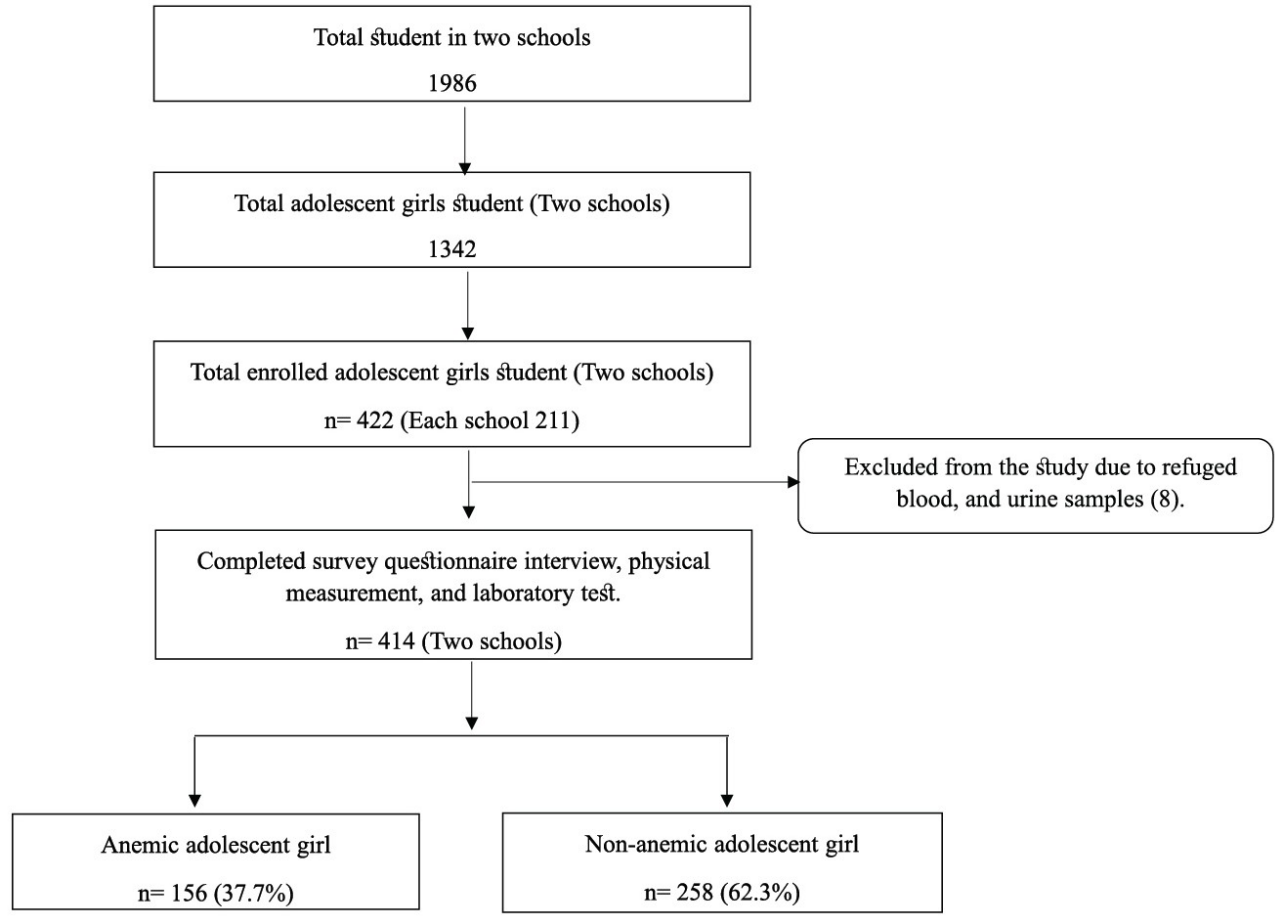
Study participant of the school-based screening for adolescent girls’ anemia in rural Bangladesh. The mean ± SD of age, BMI, MUAC, and height of the participants were 13.90 ± 1.46, 18.99 ± 3.45, 23.13 ± 2.80, and 149 ± 7.08 respectively. Of the participants, 260 (62.8%) were between the ages of 10–14 years, 258 (62.3%) were from families with ≥ 5 members, 240 (58.0%) had ≥ 3 siblings, 70 (16.9%) had illiterate mothers, 136 (32.9%) had illiterate fathers, 398 (96.1%) had mothers who were housewives, 61 (14.7%) had fathers who were farmers, and 191 (46.1%) were ≤ 10.000 BDT monthly family income. Furthermore, 95 (29.0%) of the participants using old cloth during menstruation and 73 (22.3%) experiencing irregular menstruation. Additional demographic information, hygiene maintenance, food habits, and laboratory findings are described in Table 1.

This study enrolled 422 patients from two schools, of whom 414 (98.1%) fully completed the study procedure and were thus included in the analysis (Fig 1).

#### Anemia prevalence

**Fig 2:**
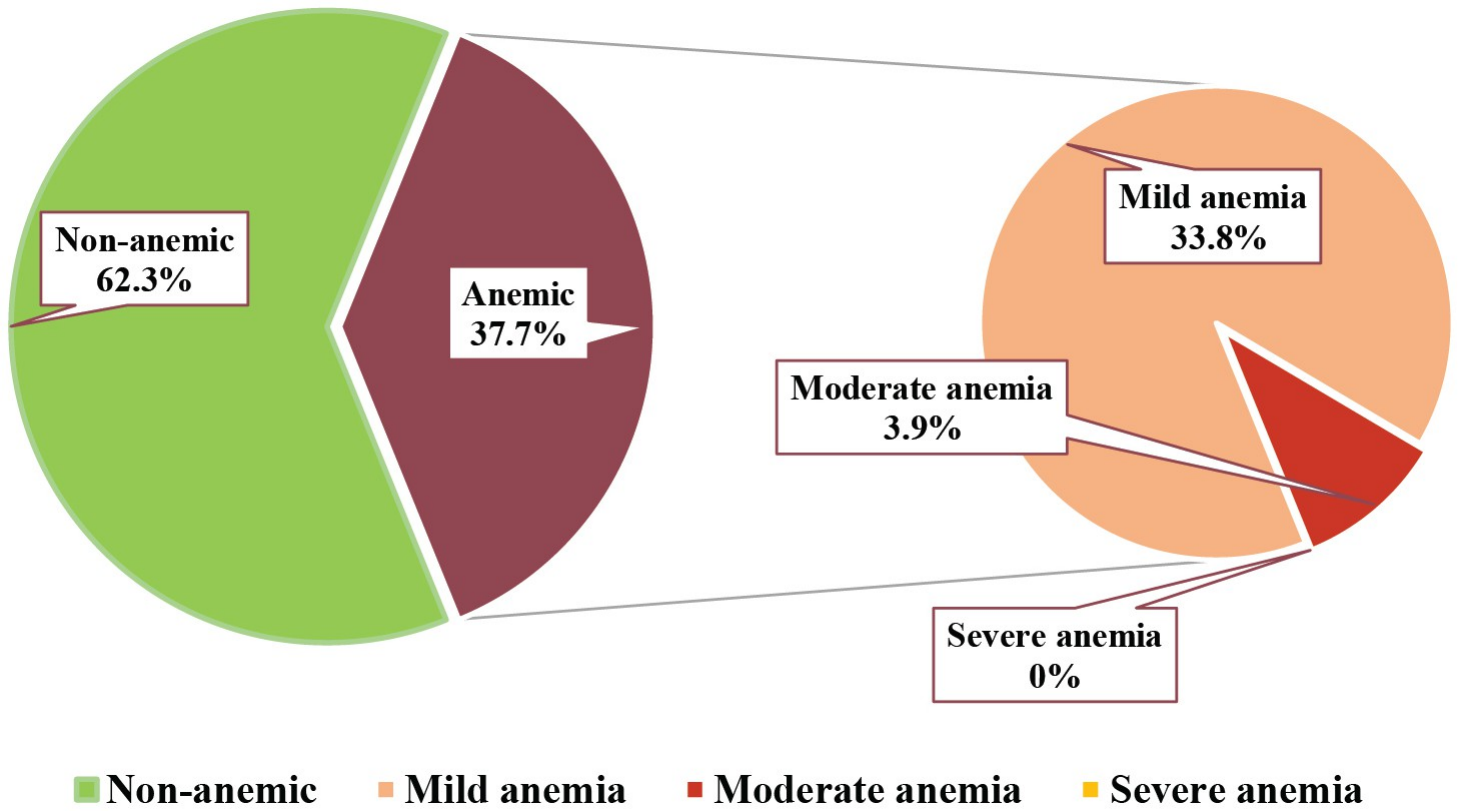
Anemia prevalence and severity of the study participants (n = 414).

In this study, the overall prevalence of anemia was 37.7% (156 cases among the screened 414 participants). Of them, 33.8% (140 cases) had mild anemia, and 3.9% (16 cases) had moderate anemia; however, there were no cases of severe anemia among the study participants (Fig 2).

#### Associated risk factors with anemia

Univariate analysis revealed that working women’s children (OR 3.84; 95% CI 1.31–11.26; *P* = 0.01), irregular lunch consumption (OR 2.15; 95% CI 1.00–4.60; *P* = 0.04), and undernutrition (OR 2.40; 95% CI 1.27–4.52; *P* = 0.01 were positively association with anemia. On the other hand, being a slab latrine user (OR 0.61; 95% CI 0.40–0.93; *P* = 0.02) and drinking pure milk not weekly (OR 0.62; 95% CI 0.41–0.92; *P* = 0.02) were negatively associated with anemia. This study did not find any significant associations between anemia and age group, student grade, parents’ education, fathers’ occupation, family income, menstruation status, frequency of menstruation, menstrual hygiene, underweight, abdominal obesity, irregular consumption of green leafy vegetables, eggs, chicken, or red meat (Table 1).

**Table 1:** Sociodemographic characteristics and risk predictors data within the anemic and non-anemic participants (n = 414).

Variable	Category	Overall n = 414 (%)	Non-anemic n = 258 (%)	Anemic n = 156 (%)	Unadjusted OR (95% CI), p—value	Adjusted OR (95% CI), p—value
Age in years (mean ±SD)		13.90±1.46	13.79±1.52	14.10±1.35	0.037	**-**
Age group	10–14 Years	260 (62.8)	168 (65.1)	92 (59.0)	0.77 (0.51–1.16) 0.21	0.68 (0.32–1.41) 0.30
	15–19 Years (Ref.)	154 (37.2)	90 (34.9)	64 (41.0)		
Schools	School A	207 (50.0)	126 (48.8)	81 (51.9)	1.13 (0.76–1.68) 0.54	1.29 (0.74–2.34) 0.37
	School B (Ref)	207 (50.0)	132 (51.2)	75 (48.1)		
Student grade	Class 6–8	240 (58.0)	149 (57.8)	91 (58.3)	1.24 (0.75–2.06) 0.40	1.89 (0.9–3.92) 0.09
	Class nine (Ref.)	91 (22.0)	61 (23.6)	30 (19.2)		
	Class ten	83 (20.0)	48 (18.6)	35 (22.5)	1.48 (0.79–2.75) 0.21	1.42 (0.69–2.94) 0.34
Family members	< 5 Members (Ref.)	156 (37.7)	102 (39.5)	54 (34.6)		
	≥ 5 Members	258 (62.3)	156 (60.5)	102 (65.4)	1.23 (0.82–1.87) 0.32	0.91 (0.36–2.29) 0.84
Sibling	≤ 2 persons (Ref.)	174 (42.0)	113 (43.8)	61 (39.1)		
	≥ 3 persons	240 (58.0)	145 (56.2)	95 (60.9)	1.21 (0.81–1.82) 0.35	1.46 (0.59–3.64) 0.41
Mother education	Literate mother (Ref.)	344 (83.1)	216 (83.7)	128 (82.1)		
	No formal education	70 (16.9)	42 (16.3)	28 (17.9)	1.13 (0.66–1.90) 0.66	1.09 (0.51–2.37) 0.82
Mother occupation	Housewife (Ref.)	398 (96.1)	253 (98.1)	145 (92.9)		
	Working women	16 (3.9)	5 (1.9)	11 (7.1)	3.84 (1.31–11.26) 0.01	7.65 (1.98–29.50) 0.00
Father education	Literate father (Ref.)	278 (67.1)	171 (66.3)	107 (68.6)		
	No formal education	136 (32.9)	87 (33.7)	49 (31.4)	0.90 (0.58–1.38) 0.63	0.70 (0.38–1.27) 0.24
Father occupation	Others (Ref.)	220 (53.1)	144 (55.8)	76 (48.7)		
	Farmer	61 (14.7)	35 (13.6)	26 (16.7)	1.41 (0.78–2.51) 0.25	0.93 (0.41–2.11) 0.86
	Business owner	133 (32.1)	79 (30.6)	54 (34.6)	1.29 (0.83–2.01) 0.25	1.23 (0.70–2.17) 0.47
Monthly family income (BDT)	≤ 10,000	191 (46.1)	118 (45.7)	73 (46.8)	0.95 (0.62–1.46) 0.82	1.18 (0.64–2.19) 0.59
	10,001–20,000 (Ref.)	165 (39.9)	100 (38.8)	65 (41.7)		
	≥ 20,001	58 (14.0)	40 (15.5)	18 (11.5)	0.69 (0.37–1.31) 0.26	0.54 (0.24–1.18) 0.12
Types of menstruation cycle	Irregularly	73 (22.3)	48 (23.9)	25 (19.7)	0.78 (0.45–1.35) 0.37	0.87 (0.47–1.60) 0.65
	Regular/Monthly (Ref.)	255 (77.7)	153 (76.1)	102 (80.3)		
Use during the menstruation cycle	Sanitary pad (Ref.)	233 (71.0)	145 (72.1)	88 (69.3)		
	Old cloth/other	95 (29.0)	56 (27.9)	39 (30.7)	1.14 (0.70–1.87) 0.58	1.36 (0.78–2.38) 0.29
Use of toilets	Modern latrine (Ref.)	160 (38.6)	89 (34.5)	71 (45.5)		
	Slab latrine	226 (54.6)	152 (58.9)	74 (47.4)	0.61 (0.40–0.93) 0.02	0.46 (0.26–0.79) 0.01
	Pit latrine	28 (6.7)	17 (6.4)	11 (7.1)	0.81 (0.36–1.84) 0.62	0.63 (0.21–1.87) 0.40
How many times a week, drink pure milk	Not weekly	195 (47.1)	133 (51.6)	62 (39.7)	0.62 (0.41–0.92) 0.02	0.65 (0.39–1.08) 0.10
	Weekly (Ref.)	219 (52.9)	125 (48.4)	94 (60.3)		
How many times a week, eat green leafy vegetables	Not weekly	62 (15.0)	44 (17.1)	18 (11.5)	0.63 (0.35–1.14) 0.13	0.64 (0.32–1.29) 0.22
	Weekly (Ref.)	352 (85.0)	214 (82.9)	138 (88.5)		
How many times a week, eat chicken	Not weekly	125 (30.2)	77 (29.8)	48 (30.8)	1.04 (0.68–1.61) 0.84	1.01 (0.55–1.83) 0.98
	Weekly (Ref.)	289 (69.8)	181 (70.2)	108 (69.2)		
How many times a week, eat red meat (Beef/ Goat)	Not weekly	324 (78.3)	204 (79.1)	120 (76.9)	0.88 (0.55–1.42) 0.61	0.79 (0.43–1.45) 0.44
	Weekly (Ref.)	90 (21.7)	54 (20.9)	36 (23.1)		
Eat breakfast	Regularly (Ref.)	372 (89.9)	234 (90.7)	138 (88.5)		
	Irregularly	42 (10.2)	24 (9.3)	18 (11.5)	1.27 (0.66–2.43) 0.47	1.21 (0.55–2.67) 0.63
Eat lunch	Regularly (Ref.)	385 (93.0)	245 (95.0)	140 (89.7)		
	Irregularly	29 (7.0)	13 (5.0)	16 (10.3)	2.15 (1.00–4.60) 0.04	2.92 (1.21–7.03) 0.02
Eat dinner	Regularly (Ref.)	301 (72.7)	191 (74.0)	110 (70.5)		
	Irregularly	113 (27.3)	67 (26.0)	46 (29.5)	1.19 (0.76–1.86) 0.44	0.94 (0.52–1.68) 0.83
BMI—Z—score	Underweight (< -2SD)	32 (7.7)	16 (6.2)	16 (10.3)	1.73 (0.83–3.58) 0.14	1.01 (0.29–3.42) 0.99
	Normal (+1 to−2 SD) (Ref.)	322 (77.8)	199 (77.1)	123 (78.8)		
	Overweight and obesity (> +1SD)	60 (14.5)	38 (14.7)	22 (14.1)	1.00 (0.56–1.77) 0.99	1.32 (0.56–3.13) 0.52
BMI (mean ± SD)		18.99±3.45	19.08±3.40	18.85±3.54	0.509	-
Hight (mean ± SD)		149.51±7.08	149.08±7.14	150.22±6.95	0.112	-
MUAC (mean ± SD)		23.13±2.80	23.25±2.65	22.92±3.02	0.245	-
MUAC	Normal (Ref.)	370 (89.4)	239 (92.6)	131 (84.0)		
	Undernutrition (10–14 years: ≤ 18.5 cm & 15–19 years old: ≤ 22.0 cm)	44 (10.6)	19 (7.4%)	25 (16.0)	2.40 (1.27–4.52) 0.01	1.43 (0.56–3.67) 0.46
Waist circumference	Normal (Ref.)	340 (82.1)	212 (82.2)	128 (82.1)		
	Abdominal Obesity (> 71 CM)	74 (17.9)	46 (17.8)	28 (17.9)	1.01 (0.60–1.69) 0.98	0.77 (0.34–1.73) 0.53

Outcome variable: Presence of anemia when non-anemia was considered as reference group (non-anemic = 0, anemic = 1).

Reference categories for independent variable were: (15–19 years, school B, class nine, < 5 members, ≤ 2 persons, literate, housewife, others, income (10,001–20,000), regular, sanitary pad, modern latrine, weekly, and normal.

T-test and multivariate logistic regression were performed. *P < 0.05.

Abbreviation: BMI = body mass index, MUAC = mid-upper arm circumference, OR = odds ratio, SD = standard deviation.

The multivariate logistic regression analysis revealed that working women (aOR 7.65; 95% CI 1.98–29.50; *P* < 0.001, slab latrine users (aOR 0.46; 95% CI 0.26–0.79; *P* = 0.01, irregular lunch consumption (aOR 2.92; 95% CI 1.21–7.03; *P* = 0.02) were associated with anemia (Table 1).

Table 2 showed no significant association between anemia and knowledge, attitude, and practice. However, when it comes to knowledge among adolescent girls, the majority were uninformed: 75.4% had not heard of anemia, 91.5% were unaware of the health risks of iron deficiency in adolescents, 92.8% were unaware of the health risks of iron deficiency in pregnant women, 80% did not know the causes of anemia, 84.8% did not know how to prevent anemia, 69.6% did not know which foods are rich in iron, 90.3% did not know which foods aid in iron absorption, and 95.9% did not know which beverages decrease iron absorption. However, the knowledge level between the anemic and non-anemic groups, a higher proportion of adolescent girls with anemia, did not know the symptoms of anemia (51.1% vs. 49.1%), did not know the causes of anemia (80.8% vs. 79.8%), did not know health risks of adolescents due to lack of iron diet (93.6% vs. 90.3%), did not know the iron-rich food (71.2% vs. 68.6%), did not know that food helps in iron absorption (90.4% vs. 90.3%), did not know that beverage decreases iron absorption (96.8 vs. 95.3%); however, a lower proportion of adolescent girls with anemia, did not hear about anemia (71.2% vs. 77.9%), did not know health risks of pregnant women due to lack of iron diet (92.3% vs. 93.0%), did not know prevention of anemia (83.3% vs. 85.7%) when compared with adolescent girls with no anemia.

**Table 2:** Association of knowledge, practice, and attitude with anemia (n = 414).

Variable	Category	Overall n = 414(%)	Anemic n = 156 (%)	Non-anemic n = 258 (%)	Unadjusted OR (95% CI), p—value
**Knowledge**					
Heard about anemia	Yes (Ref.)	102 (24.6)	45 (28.8)	57 (22.1)	
	No	312 (75.4)	111 (71.2)	201 (77.9)	0.69 (0.44–1.10) 0.12
Symptoms of anemia	Know (Ref.)	51 (50.0)	22 (48.9)	29 (50.9)	
	Don’t know	51 (50.0)	23 (51.1)	28 (49.1)	1.08 (0.49–2.39) 0.50
Health risks of adolescents due to lack of iron diet	Know (Ref.)	35 (8.5)	10 (6.4)	25 (9.7)	
	Don’t know	379 (91.5)	146 (93.6)	233 (90.3)	1.56 (0.73–3.35) 0.25
Health risks of pregnant women due to lack of iron diet	Know (Ref.)	30 (7.2)	12 (7.7)	18 (7.0)	
	Don’t know	384 (92.8)	144 (92.3)	240 (93.0)	0.89 (0.42–1.92) 0.79
Causes of anemia	Know (Ref.)	83 (20.0)	30 (19.2)	53 (20.5)	
	Don’t know	331 (80.0)	126 (80.8)	205 (79.5)	1.08 (0.66–1.79) 0.75
Prevention of anemia	Know (Ref.)	63 (15.2)	26 (16.7)	37 (14.3)	
	Don’t know	351 (84.8)	130 (83.3)	221 (85.7)	0.84 (0.48–1.44) 0.52
Know the iron rich food	Know (Ref.)	126 (30.4)	45 (28.8)	81 (31.4)	
	Don’t know	288 (69.6)	111 (71.2)	177 (68.6)	1.12 (0.73–1.74) 0.59
Food helps in iron absorption	Know (Ref.)	40 (9.7)	15 (9.6)	25 (9.7)	
	Don’t know	374 (90.3)	141 (90.4)	233 (90.3)	1.00 (0.51–1.97) 0.98
Beverage decreases iron absorption	Know (Ref.)	17 (4.1)	5 (3.2)	12 (4.7)	
	Don’t know	397 (95.9)	151 (96.8)	246 (95.3)	1.47 (0.51–4.26) 0.48
**Practice**					
Within last week, eat organ meat	Yes (Ref.)	96 (23.2)	33 (21.2)	63 (24.4)	
	No	318 (76.8)	123 (78.8)	195 (75.6)	1.20 (0.74–1.94) 0.45
Within last week, eat fresh meat	Yes (Ref.)	315 (76.1)	119 (76.3)	196 (76.0)	
	No	99 (23.9)	37 (23.7)	62 (24.0)	0.98 (0.6–1.56) 0.94
Within last week, eat fish & seafood	Yes (Ref.)	382 (92.3)	143 (91.7)	239 (92.6)	
	No	32 (7.7)	13 (8.3)	19 (7.4)	1.14 (0.55–2.38) 0.72
Within last week, eat vegetable	Yes (Ref.)	372 (89.9)	141 (90.4)	231 (89.5)	
	No	42 (10.1)	15 (9.6)	27 (10.5)	0.91 (0.47–1.76) 0.78
Within last week, eat egg	Yes (Ref.)	326 (78.7)	127 (81.4)	199 (77.1)	
	No	88 (21.3)	29 (18.4)	59 (22.9)	0.77 (0.47–1.26) 0.30
Consumption of vitamin-C-rich fruits	Yes (Ref.)	303 (73.2)	116 (74.4)	187 (72.5)	
	No	111 (26.8)	40 (25.6)	71 (27.5)	0.91 (0.58–1.42) 0.68
Everyday consumption of vitamin-C-rich fruits	Yes (Ref.)	85 (28.1)	30 (25.9)	55 (29.4)	
	No	218 (71.9)	86 (74.1)	132 (70.6)	1.19 (0.71–2.01) 0.50
Time of consumption vitamin-C-rich fruits	Before meal (Ref.)	70 (23.1)	24 (20.7)	46 (24.6)	
	During meal	14 (4.6)	8 (6.9)	6 (3.2)	2.56 (0.79–8.22) 0.12
	After meal	148 (48.8)	59 (50.9)	89 (47.6)	1.27 (0.70–2.29) 0.43
	Others	68 (22.4)	24 (20.7)	44 (23.5)	1.04 (0.51–2.10) 0.90
	Don’t know	3 (1.0)	1 (0.9)	2 (1.1)	0.99 (0.83–11.11) 0.97
Consumption of coffee/tea	Yes (Ref.)	344 (83.1)	126 (80.8)	218 (84.5)	
	No	70 (16.9)	30 (19.2)	40 (15.5)	1.29 (0.77–2.18) 0.33
Everyday consumption of coffee/tea	Yes (Ref.)	199 (57.8)	68 (54.0)	131 (60.1)	
	No	145 (42.2)	58 (46.0)	87 (39.9)	1.28 (0.82–2.00) 0.32
Time of consumption coffee/tea	Two hours/more before meal (Ref.)	14 (4.1)	4 (3.2)	10 (4.6)	
	Right before meal	81 (23.5)	29 (23.0)	52 (23.9)	1.39 (0.41–4.38) 0.60
	During meal	41 (11.9)	13 (10.3)	28 (12.8)	1.16 (0.31–4.40) 0.83
	Right after meal	112 (32.6)	45 (35.7)	67 (30.7)	1.67 (0.49–5.68) 0.41
	Two hours/ more after meal	28 (8.1)	11 (8.7)	17 (7.8)	1.62 (0.40–6.46) 0.50
	Others	64 (18.6)	24 (19.0)	40 (18.3)	1.5 (0.42–5.31) 0.53
	Don’t know	4 (1.2)	0	4 (1.8)	
**Attitude**					
How likely think to be anemic	Not likely (Ref.)	8 (1.9)	3 (1.9)	5 (1.9)	
	Not sure	373 (90.1)	134 (85.9)	239 (92.6)	0.93 (0.22–3.91) 0.93
	Likely	33 (8.0)	19 (12.2)	14 (5.4)	2.26 (0.46–11.08) 0.31
Anemia is serious	Not serious	-	-	-	-
	Not sure (Ref.)	292 (70.5)	110 (70.5)	182 (70.5)	
	Serious	122 (29.5)	46 (29.5)	76 (29.5)	1.00 (0.64–1.54) 0.99
Preparing meals with iron-rich foods	Not good (Ref.)	3 (0.7)	1 (0.6)	2 (0.8)	
	Not sure	100 (24.2)	40 (25.6)	60 (23.3)	1.33 (0.1–15.19) 0.82
	Good	311 (75.1)	115 (73.7)	196 (76.0)	1.17 (0.11–13.08) 0.90
Prepare meals with iron-rich foods	Not difficult (Ref.)	247 (59.7)	94 (60.3)	153 (59.3)	
	So-so	83 (20.0)	28 (17.9)	55 (21.3)	0.83 (0.49–1.39) 0.48
	Difficult	84 (20.3)	34 (21.9)	50 (19.4)	1.11 (0.67–1.83) 0.69
Taste of iron-rich food items or meals	Dislike (Ref.)	21 (5.1)	4 (2.6)	17 (6.6)	
	Not sure	34 (8.2)	12 (7.7)	22 (7.7)	2.32 (0.63–8.47) 0.20
	Like	359 (86.7)	140 (89.7)	219 (84.9)	2.72 (0.89–8.24) 0.08
Confident to prepare meals with iron-rich foods	Not- confident	72 (17.4)	33 (21.2)	39 (15.1)	1.35 (0.79–2.29) 0.28
	So-so	114 (27.5)	35 (22.4)	79 (30.6)	0.70 (0.44–1.14) 0.15
	Confident (Ref.)	228 (55.1)	88 (56.4)	140 (54.3)	

Univariate logistic regression was performed. *p < 0.05.

In the dietary practices among adolescent girls, in the past week, 23.2% reported consuming organ meat, while 76.1% consumed fresh meat, 92.3% consumed fish and seafood, 89.9% consumed vegetables, and 78.7% consumed eggs. Additionally, 73.2% and 83.1% reported consuming vitamin-C-rich fruits and coffee/tea, respectively. Among those who consumed these items daily, 28.1% consumed vitamin-C-rich fruits, and 57.8% consumed coffee/tea. Among all participants, 48.8% and 32.6% consumed vitamin-C rich fruits and coffee/tea after meals, respectively. When comparison practice level between the anemic and non-anemic groups, a higher proportion of adolescent girls with anemia did not eat organ meat within last week (78.8% vs. 75.6%), did not eat fish and seafood within last week (8.3% vs. 7.4%), did not consumption of vitamin-C-rich fruits (74.1% vs. 70.6%), did not drink coffee/tea (19.2% vs. 15.5%); however, a lower proportion of adolescent girls with anemia, did not eat vegetable within last week (9.6% vs. 10.5%), did not eat fresh meet within last week (23.7% vs. 24.0%), did not eat age within last week (18.4% vs. 22.9%), when compared with adolescent girls with no anemia.

Regarding the attitude of the anemia, 29.5% of participants considered it a serious condition, 75.1% believed that preparing meals with iron-rich foods is good, 86.7% liked the taste of iron-rich foods, and 55.1% felt confident in their ability to prepare iron-rich foods. When comparison attitude level between the anemic and non-anemic groups, a higher proportion of adolescent girls with anemia, did not difficult to prepare meals with iron-rich foods (60.3% vs. 59.3%), like the taste of iron-rich food items or meals (89.7% vs. 84.9%), confident to prepare meals with iron-rich foods (56.4% vs. 54.3%); however, a lower proportion of adolescent girls with anemia, did not sure how likely think to be anemic (85.9% vs. 92.6%), good in preparing meal with iron-rich foods (73.7% vs. 76.0%) when compared with adolescent girls with no anemia.

## Discussion

The overall prevalence of anemia among school-aged adolescent girls was 37.7%, which was lower than the pooled prevalence of anemia of 42.3% in Bangladesh [14]. Our study also indicated a lower prevalence of overall anemia [48.6% vs. 37.7%], mild anemia [39.2% vs. 33.8%], and moderate anemia [9.4% vs. 3.9%] when compared to data reported from the Bangladesh Demographic and Health Survey [3]. In comparison to prior research mostly conducted in urban settings, this study found a low rate of anemia, which could be attributed to improvements in healthcare facilities, education access, and parental awareness. The rural environment has a lot of fresh local iron-containing vegetables and fruits that are less expensive than in Bangladesh’s cities. However, anemia is more common among adolescent girls in our study than among women of reproductive age in Bangladesh [28]. Comparing the prevalence of anemia with our neighboring countries, we discovered a mixed picture. For instance, anemia prevalence was higher in India (56.32%) [29] and Pakistan (47.9%) [30] than in our study, while it was lower in Nepal (14.74%) [31] and Bhutan (29%) [32]. The important components of these discrepancies include the mixed picture of anemia might be due to different cultures, religions, food availability, dietary habits, and study designs. Furthermore, in our study, the prevalence of anemia among adolescent girls is substantially higher than in developed (6%) and developing countries (27%) [33]. The high frequency of anemia in developing nations like Bangladesh is caused by several causes, including diseases (such as malaria, HIV, and hookworm), unstable food supplies, and blood loss. These nations also experience a higher prevalence of anemia due to malnutrition. Anemia must be managed simultaneously with these issues to prevent long-term effects. The prevalence of anemia among adolescents in Bangladesh is decreasing, but the exact figures vary widely due to differences in criteria, settings, sample size, study population, socioeconomic variations, cultural factors, dissimilar local circumstances, assessment time, and dietary patterns across different regions.

Our finding documented a significant association between a mother’s occupation and the likelihood of her child developing anemia. Children of working mothers, in particular, were 3.8 times more likely to develop anemia than those who predominantly engaged in housekeeping responsibilities. Studies carried out in other developing countries, such as Tanzania and Brazil, came to similar conclusions [34,35]. Working mothers are less likely to give their children a balanced and sufficient diet because they find it challenging to make healthy meals because of time constraints due to their job nature [36]. Due to blanching dual responsibility, work with household responsibilities including childcare makes time constraints and limited time for cooking activities, which leads to quick and often less nutritious meal preferences. But time-efficient cooking techniques and awareness programs among other family caregivers to serve a balanced diet strategy can be effective in reducing anemia. Even while mothers often decide what their adolescents should eat in the context of rural Bangladesh, it should be a collaborative effort involving not just mothers but also caregivers or other family members to make sure a girl receives nutritious meals. Furthermore, public health intervention could be effective regarding the time-efficient balanced diet cooking techniques, time management programs, work-life balance, educational campaigns on nutrition and dietary habits to support working mothers. Although several studies have established links between anemia and the father’s occupation, parenteral education level, family income, and family size, this study could not find such links [37,38]. While other sociodemographic variables were not associated with anemia, the effect of these factors may vary based on different contextual factors.

Our study found a greater prevalence of anemia among school-aged adolescent girls who had irregular lunch consumption. In Bangladesh, students typically eat lunch on campus during school hours, either brought from home or purchased. However, some students do not eat lunch regularly or eat lunch after returning home from school. In such circumstances, they may be irregularly eating lunch, which leads to anemia. A study in Hong Kong discovered that lunch-skipping adolescents have considerably lower serum ferritin levels, indicating iron insufficiency and the onset of anemia [39]. On the other hand, while our study found no risk factors for breakfast and dinner eating habits, other studies have discovered a clear association between meal skipping and anemia [40]. The prevalence of meal skipping is higher among female than male adolescents, senior-grade students, and those living in socioeconomically disadvantaged and remote areas [41]. School-based cafeteria facilities including lunch programs can reduce the tendency to skip lunch which can be a way to prevent anemia. In order to grow and maintain their health, adolescents need to eat three meals and two wholesome snacks each day [42].

The univariate analysis results of this study demonstrated a significant association between anemia and undernutrition. Similar findings were recorded in Indonesia and India, where low MUAC levels were linked to low haemoglobin levels [43,44]. In resource-limited settings, MUAC has become popular as a test for assessing nutritional status and establishing eligibility for nutrition assistance among adults and adolescents [45]. Ensuring adequate iron, protein, and calcium intake is crucial for growth and skeletal development during adolescence. Alarmingly, over 60% of school-going adolescent girls consume less than 75% of the recommended daily allowance for iron, protein, and calcium [46], which could be a potential reason for the low MUAC levels. Unfortunately, in many developing countries, nutrition programs have primarily focused on children and women, overlooking the needs of adolescents. The long-term consequences of adolescent undernutrition include poor physical with mental development, educational performance, less productivity, anemia and during the pregnancy impaired fetal growth, pre-eclampsia, and obstructed labor that increase maternal morbidity and mortality [47]. Furthermore, they are more likely to have low birth weight babies and preterm births which lead to infant mortality, chronic health conditions and developmental delays in infants [48]. Policymakers should consider implementing school-based screening, nutrition awareness programs, and the supply of micronutrient supplements in schools. Several Studies have discovered a link between anemia and other anthropometric characteristics such as underweight, overweight, and abdominal obesity; however, no such association was identified in our study [3,32]. Therefore, screening a larger sample size will be required to acquire additional evidence.

This study discovered a protective association between anemia and adolescent girls who did not consume milk on a weekly-basis. The findings are consistent with prior research, which indicated that people who drank milk three times or more per week had a 6.9 times higher risk of being anemic than those who never drank it, not even once a week [49]. Several studies revealed with recommendation that excessive cow milk consumption led to the development of iron deficiency anemia among young children [50–53]. Milk is a good source of protein, calcium, minerals, and vitamin D, but its high casein and calcium content can hinder the absorption of iron from other foods in the digestive tract [54]. Ensuring a nutritious diet abundant in iron, vitamins, and minerals is vital for promoting the healthy growth of adolescents. We did not find any relationship between the consumption of vegetables, meat, and eggs with anemia, but research conducted in Ethiopia demonstrated that these food items act as risk predictors for adolescent girls [49]. It may be that dietary patterns varied among participants and the lack of association may indicate that anemia is influenced by a wide range of dietary components rather than specific foods.

Studies documented a protective relationship between anemia and improved sanitary toilets [55,56]. Surprisingly, this study discovered that adolescent girls who use slap latrines have a lower risk of developing anemia. We do not have an immediate explanation for this finding. Usually, poor living conditions include a variety of factors that increase the disease load, including unimproved toilets. More research with a larger sample size is needed to confirm or refute the explanation behind our data.

The KAP individual variable was not significantly associated with developing anemia among adolescent girls in this study. Our findings are supported by a study conducted in Indonesia, which concluded that KAP is not a significant factor associated with anemia in adolescent girls [11]. On the other hand, studies conducted in low- and middle-income countries like India, Malaysia, and Saudi Arabia have indicated a potential connection between knowledge, attitudes, and practices toward anemia among adolescent girls, but the findings from these studies were inconclusive [57–59]. The differences in results between our study and previous ones may be due to variations in study methodology, utilization of KAP questionnaires, and the unique characteristics of the study populations. It could be due to certain KAP factors, such as the recall questionnaire for the practice parts variable, which may have affected the appropriateness of the findings. However, the knowledge and practice levels were found to be low among the adolescent girls in our study. However, most of the participants were not well informed about anemia and its associated risk factors that could hinder their healthy growth. While overall knowledge levels were low, school-based nutrition education could be an effective means of increasing knowledge about anemia courses, consequences, and prevention among school-going adolescent girls. School-based education programs including culturally sensitive educational materials and approaches, interactive and participatory learning to engage adolescent girls, community-based interventions to engage local healthcare personnel, the wider community and parents in developing health practices are needed to increase KAP levels. This strategy can play an important role in improving knowledge levels that influence attitudes and healthy practical behaviors for better health outcomes in adolescent girls.

### Public health implications

Our study findings will contribute to enhancing public health strategies to reduce the anemia burden and slow its progression among school-going adolescent girls in rural areas of developing countries. These strategies include implementing a school-based anemia and nutritional screening program on a national scale to ensure an accurate picture of adolescent anemia including undernutrition. The study emphasizes the urgent need for public health interventions targeting working mothers to support and create an environment to ensure a balanced diet for their daughters’ well-being. Furthermore, nutritional education and school lunch program implementation can contribute to optimizing anemia reduction during this adolescent age. The aim is to provide valuable insights for decision-makers, including policymakers, economists, and technical and program staff in ministries and organizations involved in anemia interventions for public health. Moreover, the study findings are intended to inform discussions among stakeholders, helping them prioritize and tailor interventions according to the specific causes and context of anemia in their respective settings in Bangladesh and other developing nations.

### Strengths and limitations

The primary strengths of this study include the use of a standardized laboratory, the utilization of a hematological analyzer for estimating haemoglobin levels, and the presence of qualified laboratory personnel. The study’s limitations include its cross-sectional design and the inclusion of two schools, which may restrict generalizability and limited scope. To make it more generalizable, emphasize the need for further research to validate and expand upon these findings. The study relied on self-reported data through interviews, which may be subject to recall bias or social desirability bias. In addition, our study is attributed to the undernutrition status solely assessment through MUAC measurements and the recall questionnaire for practice part, may influenced the appropriate finding of KAP.

## Conclusions

The study findings revealed a high rate of anemia prevalence among school-going adolescent girls in rural areas, in which having a working mother, irregular lunch consumption, and undernutrition played a significant role toward anemia, while weekly milk consumption and the use of slab latrines were found to be protective factors. This research is a valuable resource for policymakers seeking to improve adolescent health in rural areas of developing countries, even if more studies with a more robust research design, a broader and more diverse sample size is warranted to elucidate the underlying mechanisms and validate these findings. Therefore, we recommend implementing interventions such as school-based anemia screening programs, nutritional health education, and the addition of lunch with iron supplementation to the national education curriculum aimed at curbing anemia on a larger scale. Additionally, a community-based nutrition awareness program with focus on working women should be implemented. These strategies aim to effectively prevent and control anemia in adolescent girls.

## Supporting information

S1 File Global research questionnaire. (DOCX)
